# Assessing healthcare access using the Levesque’s conceptual framework– a scoping review

**DOI:** 10.1186/s12939-021-01416-3

**Published:** 2021-05-07

**Authors:** Anthony Cu, Sofia Meister, Bertrand Lefebvre, Valéry Ridde

**Affiliations:** 1 Department of Health, Republic of the Philippines, Manila, Philippines; 2 Centre Population et Développement (Ceped), Institut de recherche pour le développement (IRD) et Université de Paris, Inserm ERL 1244, 45 rue des Saints-Pères, 75006 Paris, France; 3 University of Rennes, EHESP, CNRS, ARENES – UMR 6051, F-35000 Rennes, France

**Keywords:** Scoping review, Healthcare access, Levesque’s framework, Health system

## Abstract

**Introduction:**

Countries are working hard to improve access to healthcare through Universal Healthcare Coverage. To genuinely address the problems of healthcare access, we need to recognize all the dimensions and complexities of healthcare access. Levesque’s Conceptual Framework of Access to Health introduced in 2013 provides an interesting and comprehensive perspective through the five dimensions of access and the five abilities of the population to access healthcare. The objectives of this paper are to identify and analyze all empirical studies that applied Levesque’s conceptual framework for access to healthcare and to explore the experiences and challenges of researchers who used this framework in developing tools for assessing access.

**Methods:**

A scoping review was conducted by searching through four databases, for studies citing Levesque et al. 2013 to select all empirical studies focusing on healthcare access that applied the framework. An initial 1838 documents underwent title screening, followed by abstract screening, and finally full text screening by two independent reviewers. Authors of studies identified from the scoping review were also interviewed.

**Results:**

There were 31 studies identified on healthcare access using the Levesque framework either a priori*,* to develop assessment tool/s (11 studies), or a posteriori*,* to organize and analyze collected data (20 studies)*.* From the tools used, 147 unique questions on healthcare access were collected, 91 of these explored dimensions of access while 56 were about abilities to access. Those that were designed from the patient’s perspective were 73%, while 20% were for health providers, and 7% were addressed to both. Interviews from seven out of the 26 authors, showed that while there were some challenges such as instances of categorization difficulty and unequal representation of dimensions and abilities, the overall experience was positive.

**Conclusion:**

Levesque’s framework has been successfully used in research that explored, assessed, and measured access in various healthcare services and settings. The framework allowed researchers to comprehensively assess the complex and dynamic process of access both in the health systems and the population contexts. There is still potential room for improvement of the framework, particularly the incorporation of time-related elements of access.

**Supplementary Information:**

The online version contains supplementary material available at 10.1186/s12939-021-01416-3.

## Background

Healthcare access has been improving continuously throughout the world in the last decades. The Global Burden of Disease Study reported sustained increase in the Global Healthcare Access and Quality Index Scores: from 37.6/100 in 1900 to 42.4 in 2000 and 54.4 in 2016 [1]. However, access to health care remains a major problem, despite the adoption of universal healthcare coverage (UHC) by member countries of the World Health Organization (WHO) [2]. Around 7.3 billion people are unable to access all the essential health services that they need, according to the 2017 Global Monitoring Report [3]. A systematic analysis of amenable deaths in 137 countries estimated that around 8.6 million excess deaths occurred in 2016 as a consequence of problems in access or quality of healthcare, particularly in Low and Middle-Income Countries (LMICs) [4].

Healthcare access is a complex concept closely intertwined with health systems performance [5, 6]. The WHO has been pushing its member countries to implement health sector reform geared towards the achievement of UHC. Access to healthcare in the UHC context has given much emphasis on the financial aspect of access. The WHO defines UHC as “*ensuring that all people have access to needed health services (including prevention, promotion, treatment, rehabilitation, and palliation) of sufficient quality to be effective while also ensuring that the use of these services does not expose the user to financial hardship”* [7]. However, when talking about healthcare access, in addition to affordability, there are other dimensions or abilities that merit attention [8, 9].

### Different access frameworks

There are numerous frameworks for healthcare access, each with its own merits and weaknesses [6]. One of the most extensively used access framework is the Andersens’ Behavioural Model of Health Services Use, which views access as a function of health services use predisposition, healthcare need, enabling and impeding factors to utilization [10]. In Penchansky and Thomas’ framework, access is portrayed as a “fit” between the needs of patients and the capacity of healthcare systems [11]. Frenk’s framework is another commonly cited framework that defines access as the population’s ability to seek then obtain care. Frenk’s framework further identifies the availability of resources, utilization power, and resistance as the dimensions of access as well as assess the performance of health systems [12]. Many other definitions and frameworks of healthcare access exist, however, one of the most comprehensive and recent is Levesque’s Conceptual Framework for Healthcare Access [6].

### The conceptual framework of access to healthcare by Levesque et al.

**Fig. 1:**
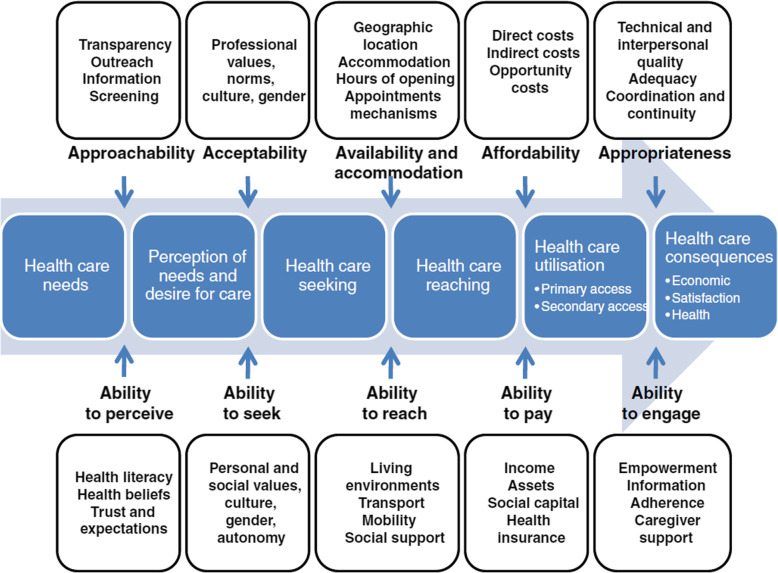
Levesque conceptual framework for healthcare access

The Conceptual Framework of Access to Healthcare by Levesque et al. was published in 2013 and was developed as a result of a comprehensive review of existing literature on healthcare access [6] (Fig. 1). The framework suggests a multidimensional view of healthcare access in the context of health systems with dimensions of approachability, acceptability, availability/accommodation, affordability, and appropriateness. It takes into account the population’s socioeconomic determinants resulting in the incorporation of the five corresponding abilities of individuals and populations: to perceive, to seek, to reach, to pay, and to engage, in healthcare [6]. The framework is able to take into equal account both the health systems and the patient’s perspective with regard to access. The framework allows researchers to look into barriers to access that happen as a consequence of people’s ability to perceive, seek, reach, pay or engage [13] and not just the failures of the health system. Levesque’s framework defines access as the opportunity to identify, seek, reach, obtain, or use healthcare and to ensure the fulfillment of the needs for these services [6].

It has been almost 7 years since the Conceptual Framework of Access was published. The framework has been gaining wide use in research about healthcare access. With its increasing use, there is a need to conduct a study on how the framework has been used and how well it has worked for researchers studying healthcare access. This paper aims to conduct a scoping review of studies that assessed healthcare access that applied the Levesque framework. The objectives of this paper are: 1) To identify and analyze all empirical studies that utilized Levesque’s conceptual framework for access to healthcare either a priori, to develop data collection tools or a posteriori, to arrange and analyze collected data; and 2) To explore the experiences of researchers in the use of the Levesque framework in developing tools for assessing access or in organizing and analyzing collected data on access to healthcare.

## Methods

The conduct of the scoping review was guided by the VERDAS consortium generic protocol and PRISMA-ScR (refer to Additional file 1 for the PRISMA-ScR checklist). The protocol consists of six stages: (a) defining the research question; (b) identifying relevant studies or search strategy; (c) selecting studies; (d) charting the data and assessing the quality of studies included; (e) collating, summarizing, and reporting the data; and (f) consultation [14] (Fig. 1).

### Definition of the research question

The research question in this scoping review is: *How has the Levesque framework been used in empirical studies assessing healthcare access?*

### Identification of relevant studies

Several scientific and grey literature databases, namely Pubmed, Scopus, Web of Science, and Google Scholar were searched to identify all studies citing the article: *Patient-centred access to health care: conceptualising access at the interface of health systems and populations*, by Levesque, J.F. et al., which introduced the framework in 2013. All the identified studies from April 2013 to January 2020 were saved in Zotero® reference manager software.

#### Eligibility criteria

Inclusion and exclusion criteria were defined post-hoc through pilot screening of 20 randomly chosen studies by AC with SM. The following were the final inclusion criteria developed: (a) The terms “access*” OR “approachability” OR “acceptability” OR “affordability” OR “availability” OR “appropriateness” OR “abilit*” OR “accessibilité” OR “acceptabilité” OR “disponibilité” OR “adaptation” OR “appropriation” AND “health” OR “healthcare” OR “santé” OR “soins de santé”, were used in the title of the study. These specific terms were chosen for the inclusion criteria as they are the exact terminologies in the Access to Healthcare framework by Levesque et al.; (b) Access to healthcare was the main focus of the study; and (c) The Healthcare Access Framework by Levesque et al., was utilized either a priori, for the development of data collection tool/s or a posteriori, to organize and analyze collected data. Articles were excluded if they were (a) written in other languages other than English and French; b) a non-empirical study; or (c) not available as a full article.

### Selection of studies

The initial list of 1838 identified studies showed many duplicates, which is a good indication of the completeness of the search. Duplicates were then removed as well as those written in languages other than English and French. Title screening was conducted using the identified key terminologies stated in the inclusion criteria (a). Then, non-empirical studies were identified and excluded from the list through the Automated Text *Classification of Empirical Records (ATCER).* ATCER is an online tool that allows for the automatic categorization of publications indexed in bibliographical databases into empirical and non-empirical studies to help researchers in conducting scoping or systematic reviews [15].

The program classifies studies as “empirical” when the calculated percentage is 50% or higher and “Non-empirical” when the percentage is less than 50%. The entire process was conducted by CR, who is affiliated with the University of Montreal which developed ATCER. As an additional quality control measure, 21 studies whose percentage of probability as an empirical study was rated between 40 and 60% were also manually revisited to ensure the correctness of the automated categorization. The abstract review was done to filter only those studies where healthcare access or its dimensions were the main focus.

All 121 studies that met the inclusion criteria in the title and abstract screening underwent full article review. Full article review was done to identify studies that utilized Levesque’s Conceptual Framework of Access to Healthcare either in the development of its data collection tool/s or in organizing and analyzing collected data. A second reviewer, VR, was consulted whenever difficulty or questions arise during any stage of the title screening, abstract screening, and full article review.

### Data collection and study quality assessment

A data extraction matrix using Excel® was used to collect both macro descriptive data such as author/s, title, year of publication, type of study, and microdata such as the number of respondents, study design, the geographical scope of the study (local, national, international), study country setting (low- and middle-income country vs high-income country), type of data analysis used, how Levesque framework was used (a priori vs a posteriori), data collection tools used, focus on access (health systems vs individual) and the dimensions/abilities of access explored.

### Data synthesis and reporting

Questions pertaining to access to healthcare were collected from all the tools (individual interview guide, focus group discussion guide, questionnaires, surveys, etc.) used in each of the studies included in the final list of this scoping review. A collation matrix was developed to clearly show the following information about each of the questions, such as type of health service, type of question (qualitative vs. quantitative), target respondent (recipient/patients vs. health providers), and study country setting (LMIC vs. HIC).

Further, for each question collected, the specific dimension, ability, and sub-dimension based on the Levesque framework were identified. To achieve this, the original dimension/ability categorization of each question (whenever such categorization was available in the study tool) was included in the matrix. In addition, two independent researchers, AC and SM with strong familiarity regarding the Levesque framework were tasked to categorize each question into a specific dimension/ability and to identify the appropriate sub-dimension. A third researcher, SL, who had previously worked on the framework was consulted to settle any categorization discrepancies.

### Qualitative interviews (consultations)

A qualitative interview of the authors of these studies was conducted. A semi-structured interview guide (Additional file 1) was created to explore how they used the framework and gain insight into their experience and the challenges they have encountered in the use of the framework. Each of the main authors or corresponding authors of the studies identified in the scoping review was contacted by email available in the published article. Those who responded and agreed to an interview were sent an Interview Consent Form for signature and an interview schedule was set. The interview was conducted online. The semi-structured interview lasted between 20 to 30 min and was audio-recorded with permission. The interviews were then transcribed and anonymized and then analyzed through framework analysis [16].

## Results

### Search findings and study selection

**Fig. 2:**
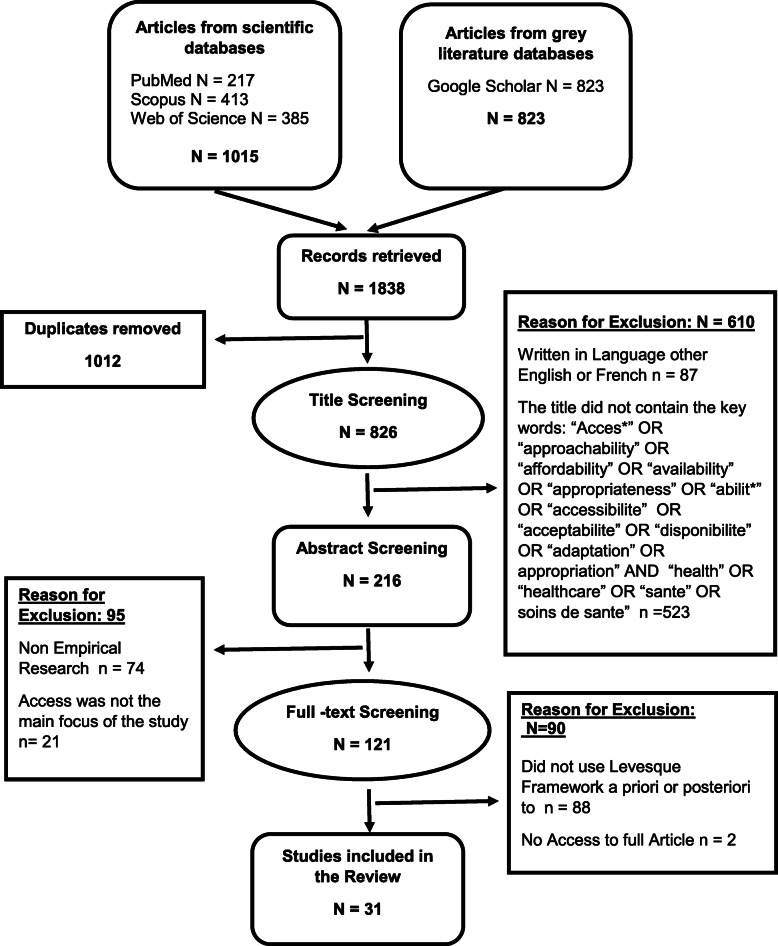
Study selection process PRISMA diagram

Results from the database search and study selection processes are shown in PRISMA diagram (Fig. 2) below. A final list of 31 studies met all the eligibility criteria .

### Study general characteristics

Among the 31 articles identified, 27 (87%) were published in different scientific journals while 4 of them were theses or dissertations. Fifteen or almost half of the studies were qualitative types [17–31]. These qualitative studies explored access to various maternal and child healthcare services [17, 19, 22–24, 28, 31] or access to health services of different groups of people such as refugees [17, 23, 26, 32], elderly [21], differently-abled people [25], LGBTQ [27], and migrant minorities [30, 33]. On the other hand, there were eight quantitative descriptive studies [13, 34–40]. These quantitative studies attempted to measure access to school health services [35], mental healthcare services [36, 37, 39, 40], and primary healthcare services [13, 34, 38]. The remaining eight were mixed-method studies [41–48] which included two studies that looked into access to primary healthcare [42, 43]; one study on maternal healthcare [44]; three studies on access to healthcare of vulnerable and indigenous populations [41, 45, 46]; and two which attempted to develop measures for the specific dimensions of access such as affordability, availability, and accommodation [47, 48]. It is also interesting to note that aside from studies that assessed access to healthcare, there were also those that identified, explored, or measured different barriers to access [13, 21, 31, 32, 36, 38, 44].

The number of respondents in qualitative studies, which range from six to 105, is understandably smaller compared to the respondents count for the quantitative and mixed-method studies. The qualitative interviews with a lower number of respondents are those which aimed to explore access or perceptions to access of a particular group of people, such as pregnant refugees [23], transgender persons with HIV [27], and caregivers of children with cerebral palsy [25]; Qualitative studies that had comparatively more respondents were those which also conducted focused group discussion in addition to one-on-one interviews [18, 24, 26, 28, 30, 45]. With regard to quantitative studies, the number of respondents ranged from 372 to 27,580. It is important to note that the quantitative studies that had a very large number of respondents used secondary data. That is, they used existing data collected from national or international studies on health and healthcare [13, 34, 36, 38–40].

**Fig. 3:**
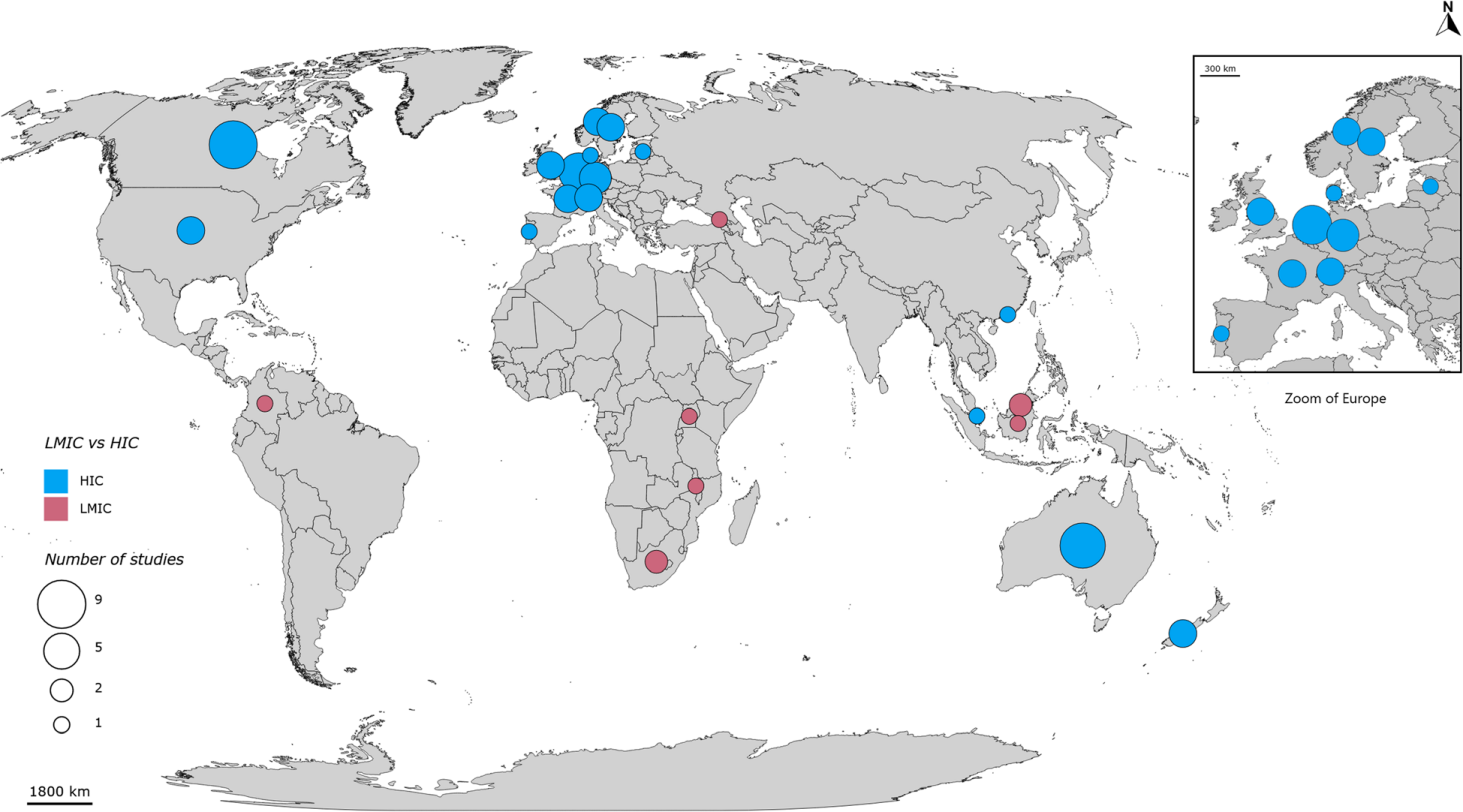
Geographic distribution of identified studies

With regard to country settings, 22 (71%) of the studies were conducted in high-income countries (HIC) [13, 21, 23, 26, 30, 31, 33, 35–48], and only nine (29%) were conducted in low-and middle-income countries (LMIC) [18, 19, 22, 24, 25, 27, 28, 32, 34]. Figure 3 shows the geographic distribution of identified studies most of which were conducted in HICs. Lastly, there were studies conducted in local (14 studies), regional (two studies) and national scales (10 studies), while five were conducted on international or multiple countries scale.

### Use of Levesque access to healthcare framework

#### A priori Vs a posteriori use of the framework

The use of Levesque access to healthcare framework among the 31 identified studies in the scoping review can be classified into either a priori or a posteriori*.* There were 11 studies that used the framework a priori to develop its data collection tools such as interview guides, focus group discussion guides, and questionnaires [25–28, 30, 33, 35, 37, 44, 47–49]. The other 20 studies applied the framework in organizing and analyzing collected data [13, 17–19, 21–24, 31, 32, 34, 36, 38–43, 45, 46]. It is also interesting to note that some studies [13, 38–40, 50] utilized secondary data from past surveys, which were not originally designed for the Levesque framework. Additional file 2 provides a summary of characteristics of the 31 identified studies.

#### Partial use of the framework

While all the studies identified in the scoping review utilized the Lévesque framework, only 11 of them explored both the dimensions and the abilities of access to healthcare [17, 19, 24, 26, 27, 31, 32, 37, 41, 45, 46]. One of the main strengths of the Levesque framework is that it takes into account both the health systems perspective of access through its dimensions and the population’s/patient’s perspectives on access through their abilities of access. However, instead of looking at both, several studies chose to only focus on the dimensions of access or on the abilities of access. Out of the 31 studies,13 focused solely on the health systems dimensions of healthcare access [13, 21, 28, 33, 35, 38–40, 42–44, 47–49] while seven focused only on population abilities aspect of access [18, 22, 23, 25, 30, 34, 36]. Table 1 provides summarized details on the use of the Levesque framework and the dimensions/abilities explored or measured in each of the 31 studies.

**Table 1:** Summary of use of Levesque framework and dimensions/abilities explored

	Author	Year	How Levesque framework was used	Access focus (Dimensions VS Abilities)	Number of dimensions /abilities Explored	Dimensions and abilities explored YES/NO)									
						Approachability	Acceptability	Availability/ accommodation	Affordability	Appropriateness	To perceive	To seek	To reach	To pay	To engage
1	Abdelwahab	2017	A Posteriori	BOTH	10	Yes	Yes	Yes	Yes	Yes	Yes	Yes	Yes	Yes	Yes
2	Bailie	2015	A Posteriori		10	Yes	Yes	Yes	Yes	Yes	Yes	Yes	Yes	Yes	Yes
3	Chuah	2018	A Posteriori		10	Yes	Yes	Yes	Yes	Yes	Yes	Yes	Yes	Yes	Yes
4	Fathi Afshar	2019	A Priori		10	Yes	Yes	Yes	Yes	Yes	Yes	Yes	Yes	Yes	Yes
5	Fauk	2019	A Priori		10	Yes	Yes	Yes	Yes	Yes	Yes	Yes	Yes	Yes	Yes
6	Matthews	2019	A Posteriori		10	Yes	Yes	Yes	Yes	Yes	Yes	Yes	Yes	Yes	Yes
7	Richard	2019	A Posteriori		10	Yes	Yes	Yes	Yes	Yes	Yes	Yes	Yes	Yes	Yes
8	Russell	2016	A Posteriori		10	Yes	Yes	Yes	Yes	Yes	Yes	Yes	Yes	Yes	Yes
9	Viveiros	2018	A Posteriori		10	Yes	Yes	Yes	Yes	Yes	Yes	Yes	Yes	Yes	Yes
10	Ward	2018	A Posteriori		10	Yes	Yes	Yes	Yes	Yes	Yes	Yes	Yes	Yes	Yes
11	Corscadden	2018	A Posteriori	DIMENSIONS	5	Yes	Yes	Yes	Yes	Yes	No	No	No	No	No
12	Corscadden	2019	A Posteriori		5	Yes	Yes	Yes	Yes	Yes	No	No	No	No	No
13	Corscadden	2017	A Posteriori		5	Yes	Yes	Yes	Yes	Yes	No	No	No	No	No
14	Corscadden	2018	A Posteriori		5	Yes	Yes	Yes	Yes	Yes	No	No	No	No	No
15	Miteniece	2018	A Priori		5	Yes	Yes	Yes	Yes	Yes	No	No	No	No	No
16	Miteniece	2019	A Priori		5	Yes	Yes	Yes	Yes	Yes	No	No	No	No	No
17	McDonald	2015	A Posteriori		5	Yes	Yes	Yes	Yes	Yes	No	No	No	No	No
18	Abduludin	2019	A Priori	ABILITIES	5	No	No	No	No	No	Yes	Yes	Yes	Yes	Yes
19	Anstey Watkins	2019	A Posteriori		5	No	No	No	No	No	Yes	Yes	Yes	Yes	Yes
20	Gomez	2020	A Posteriori		5	No	No	No	No	No	Yes	Yes	Yes	Yes	Yes
21	Packness	2019	A Posteriori		5	No	No	No	No	No	Yes	Yes	Yes	Yes	Yes
22	Vandan	2019	A Priori		5	No	No	No	No	No	Yes	Yes	Yes	Yes	Yes
23	Gordon	2017	A Posteriori		5	No	No	No	No	No	Yes	Yes	Yes	Yes	Yes
24	Celik	2016	A Posteriori	VARIOUS	4	No	No	No	Yes	Yes	Yes	Yes	No	No	No
25	Doetsch	2017	A Posteriori		4	Yes	Yes	Yes	Yes	No	No	No	No	No	No
26	Tam	2017	A Priori		4	Yes	Yes	Yes	No	Yes	No	No	No	No	No
27	Henry	2020	A Posteriori		3	No	No	No	No	No	Yes	Yes	No	No	Yes
28	Bezem	2017	A Priori		2	No	No	Yes	Yes	No	No	No	No	No	No
29	Roberge	2014	A Priori		2	Yes	No	No	No	No	No	No	Yes	No	No
30	Haggerty	2017	A Priori		1	Yes	No	No	No	No	No	No	No	No	No
31	Haggerty	2015	A Priori		1	No	Yes	No	No	No	No	No	No	No	No

### Characteristics of tools used

The identified studies used different and sometimes multiple tools to collect data on healthcare access. Structured interviews were used in 16 of the studies, focus group discussion guides were used in eight, in-depth interview guides in three, and survey questionnaires in six. There was one study that also used non-participant observation as one of its multiple data collection tools. On the other hand, there were six studies that used existing data collected from prior national or international surveys. It is important to note that while the type of data collection tool used in each study was clearly stated in every article, not all data collection and assessment tools were readily available and some were not retrieved despite efforts.

From these tools, 62 quantitative and 85 qualitative questions, for a total of 147 unique questions on access to healthcare were extracted (Additional file 3). These questions covered a wide variety of types of health services including general/primary health care services, maternal and child care, oral and dental health care, HIV/AIDS and infectious diseases, mental health, and school health services. Relatively half (51%) of the questions were concerned with general/primary health care. Table 2 provides a summary of the extracted qualitative and quantitative questions on access categorized by type of health services.

**Table 2:** Summary of extracted qualitative and quantitative questions on access categorized by type of health services

Types of services	Quantitative	Qualitative	Total
General/ Primary Health Care Services	42	33	**75**
Maternal and Child Care	5	31	**36**
Infectious Diseases / HIV/AIDS Health Services	0	21	**21**
Oral and Dental Health	1	0	**1**
Mental Health	4	0	**4**
School Health Services	10	0	**10**
**Total**	**62**	**85**	**147**

Although all dimensions and abilities of access based on Levesque’s framework were represented in the collected questions, some were more represented than others. Among the dimensions of access, appropriateness was the most represented with 33 questions, followed by affordability and availability/accommodation having 21 questions each. Acceptability and approachability were the least represented access dimensions with seven and nine questions, respectively. As for the five Levesque’s population/individual abilities of access, the most represented was the ability to perceive with 22 unique questions, while the least represented was the ability to engage with six questions. Table 3 provides a summary of extracted qualitative and quantitative questions categorized by dimensions and abilities based on the Levesque framework. Concerning target respondents, 108 questions (73%), an overwhelming majority, were designed to be answered by recipients (patients, clients, or community members). Questions designed for health providers numbered 29 (20%) while the remaining 10 (7%) were addressed to both health providers and recipients. Table 4 provides a summary of extracted qualitative and quantitative questions categorized by target respondent.

**Table 3:** Summary of extracted qualitative and quantitative questions categorized by dimensions and abilities

Dimensions/abilities	Quantitative	Qualitative	Total
Approachability	5	2	**7**
Acceptability	2	7	**9**
Availability/Accommodation	11	10	**21**
Affordability	10	11	**21**
Appropriateness	25	8	**33**
To Perceive	2	20	**22**
To Seek	0	8	**8**
To Reach	3	7	**10**
To Pay	0	10	**10**
To Engage	4	2	**6**
**Total**	**62**	**85**	**147**

**Table 4:** Summary of extracted qualitative and quantitative questions categorized by target respondent

Respondent	Quantitative	Qualitative	Total
Health Providers	11	18	**29**
Recipients	50	58	**108**
Both	1	9	**10**
**Total**	62	85	**147**

### Interview findings

While there was a total of 31 eligible studies, only 26 (lead) authors needed to be contacted since some studies had the same author. Out of the 26 authors that needed to be contacted, only 21 authors had up-to-date contact details. Five studies did not provide the authors’ contact details, particularly dissertations or theses, or the provided contact details were no longer relevant. Furthermore, of the 21 invited authors, only nine (43%) responded for an interview. Second and third follow-up emails were also sent to authors who did not respond to the initial email, a week and 2 weeks later, respectively. In the end, a total of seven interviews were successfully conducted. The seven interviews represented 11 studies as one of those interviewed, authored four studies and another authored two studies, resulting in 35% coverage.

Four main themes were identified during the analysis of the interviews: (a) reason for using Levesque framework; (b) Framework use experiences; (c) measures taken to address challenges, and (d) recommendations for future studies on access.

#### Reason for the use of Levesque framework

Various reasons were given by respondents for their choice in using Levesque’s conceptual framework for access to healthcare over other access frameworks. The most common among these are the authors’ perceptions that the Levesque framework is currently the most comprehensive framework for access or an improvement from existing ones. This particular perception is grounded on the knowledge that a comprehensive review of literature served as the basis for the development of the framework.

*“When I looked at the actual publication, one of the big strengths was what a big review of literature was done to base the original framework on … . It synthesizes the different literatures that had looked into access to care and it incorporated different conceptualization on what access means”*. – Author 03

Another common reason provided regarding the choice of the framework was that the Levesque framework considers both health systems or health provider’s perspective and patient’s or client’s perspective on access. While other access frameworks also look at access as a function of supply and demand, the Levesque framework incorporates the parallel element of abilities of individuals or populations. This factor seemed to have been a major factor in the choice of researchers who in particular wanted to explore access from the perspective of the patients. One author even emphasized that the concept of abilities of communities was what drove their team in the use of this framework.

As for a number of other respondents, the choice was due to how the Levesque framework shows access as a process or a journey instead of a static concept as others define it.

*“It prioritizes the balance between the services delivery, the wants, needs, and the access journey in a sense … . it’s got an active nature to it, so it reflects particularly the active journey that community members take on, in seeking care.” – Author 05*

*“It acknowledges that you haven’t just accessed care once you got into the door … the (Levesque) framework’s looks at access as a process from before getting to the door until the end of treatment” – Author 06*

Other cited reasons for the use of the framework include: prior familiarity with the framework, use of the framework in a previous study, worked with colleagues that have previously used the framework, previously worked with one of the co-authors of the framework, and one even admitted candidly that being one of the co-authors of the framework played a role in the choice of its use.

#### Experiences in the use of the framework

Some researchers highlighted that the framework was easy enough to operationalize:

*“This framework is more process-oriented and thus easy to operationalize for the research … it was a very explanatory and directing model” – Author 02*

It is important to note that for those who had used the Levesque framework a priori*,* the framework made it easier to ensure that different aspects of the process of access were looked into. It is of particular value to those who explored the concept of barriers to access. In such studies, it is important to look at all the possible aspects of access, and their corresponding barriers and their interactions. On the other hand, those that used the framework a posteriori also had positive experiences such as findings fitting well with the framework that made them choose to use the Levesque framework in the first place or feel validated in their choice of framework.

*“We started with an open coding process. We started grouping the key findings of the various studies that were included in the scoping review into themes and categories. Then, when we looked at that, we actually realized that it actually fitted very nicely within the Levesque framework. So, we actually applied the Levesque framework after we have done the initial open coding of the data.” - Author 03*

As for the challenges experienced, the most commonly cited were repeated instances where there were difficulties in categorizing questions or responses into specific abilities or dimensions. There were also instances that responses would fall into more than one dimension or ability. As an example, it was pointed out that one patient’s response to barriers to access concerning health facilities is that the facilities are located too far away from the community. The response could either be placed under the availability dimension if one looks at it in the context of geographical distance, or under the dimension of ability to pay if the cost of transportation was instead considered. Another example is the courtesy and respect (or the lack thereof) given by healthcare workers when providing services, which can either be categorized as appropriateness if one chooses to look at it as an issue of interpersonal quality of care by health workers, or as acceptability if one chooses to look at it as an issue of the patients’ value with regards to possible treatment at the health facilities.

*“I think that it was a challenge, some of the dimensions blurred with each other. Things like affordability and ability to pay are quite straight forward, but when you get down to some of the other dimensions it can a little bit difficult to sort of tell … ” Author 05*

Another common challenge is that there is a notable imbalance in the representation of the different dimensions and abilities of access. In particular, availability, affordability, and especially appropriateness are more frequently taken into account by both health providers and patients when talking about access. This particular imbalance is most prominently noted by authors who worked on studies that utilized existing (secondary) data. Discussions also arose with regards to the appropriateness as a measure of quality instead of access. However, the author who raised the challenge mentioned that the team eventually agreed to follow the Levesque framework which views it as a dimension of access.

*“ … when we have mapped probably around 50 questions from the survey to these five domains, a majority of them fell in the appropriateness domain because it was so broad. It’s about communication. It’s about what happens after you’ve reached care essentially.” – Author 06*

#### Measures taken to address experienced challenges

The authors shared some of the measures they have taken to address the challenges they have experienced in the use of the framework. With regard to the difficulty in categorizing into a specific dimension/ability of access, one author shared that it was necessary to ensure that team members use the same conceptual definitions by consulting the original paper by Levesque et al.

*“I think what we often did is that we went back to the paper. I think, the definition in the paper was actually pretty good … So we would go back there regularly just to be sure that we were clear about it, that everyone was speaking the same language.” – Author 05*

Another strategy to address confusion in categorization is to have at least three different individuals familiar with Levesque framework for access, independently categorizing the information. As for the challenge of imbalance in the representation, awareness of the different access dimensions and abilities when designing data collection tools allow this concern to be immediately addressed.

## Discussion

### Use of Levesque’s conceptual framework for access to healthcare

The conceptual framework for healthcare access by Levesque et al. was published almost 7 years ago but still can be considered a relatively new framework on healthcare access. While there is a multitude of articles and conceptual frameworks that attempts to define the complex concept of access and its dimensions [51], the result of this scoping review shows that the Levesque framework has been gaining acceptance among experts conducting research in this field. The fact that the framework was developed through an extensive review of literature on access, and that it takes into account both the health systems and population perspective on access [6] was recognized and appreciated by researchers who decided to use it especially those that developed their data collection tools based on the framework. For researchers that collected data beforehand, they have found that almost all their findings would fit within the different dimensions and abilities of access in the Levesque framework. It would be not have been possible for some other more restrictive frameworks on healthcare access. The result of the interviews with the authors showed positive overall experience in empirically using the framework that they would most likely use the same framework for their future research on healthcare access should the opportunity arise. One author even described the Access to Healthcare Framework as looking at access dimensions as process-oriented and thus easy to operationalize for the research team.

### Challenges in the use of the framework

While the overall experience of most authors is positive, they have also shared some challenges in the use of the framework. The most commonly experienced challenge in the use of the framework is the difficulty in categorizing certain questions or data into a specific dimension or ability of access. This difficulty was also observed during the scoping review when questions from the different tools used in the identified studies were collated and some were found to have been potentially miscategorized. The inherent complexity of the concept of access makes it difficult to clearly delineate some dimensions and abilities of access. Some healthcare access questions might not necessarily fall into a single dimension or ability. A single question can be used to assess or explore two or more dimensions or abilities of access. As such it might be possible to decrease the number of questions in assessment tools. However, for studies assessing or exploring specific dimensions or abilities of access, it is necessary to pay close attention to the wording and framing of the questions in order to elicit appropriate response.

Another potential weakness of the framework that was identified is its inability to take into account time-related elements of access. The availability/accommodation dimensions of the framework include sub-dimensions such as geographic location, accommodation, opening hours, and appointment mechanism. However, some studies [13, 27, 38–40, 44] in this scoping review identified questions and data on access referring to patient waiting time and travel time that is not necessarily a consequence of distance. These questions could not be easily categorized in any of the framework’s dimensions or abilities. Geographic accessibility can be measured in geographical or Euclidean distance and in time-distance, and as such is only partially covered by Levesque framework. It might be worth to consider including time-related elements of access such as travel time. The results of this scoping review also shows that, less research on access to healthcare was conducted in LMICs. This is a cause for concern considering that problems in healthcare access inequity are more often experienced in LMICs as shown by the lower Healthcare Quality and Access (HAQ) index in these countries [52]. The conduct of more research on healthcare access in LMIC settings should be pursued. Information and knowledge gained from LMIC research on access would be essential to address healthcare inequity in these countries. Since this scoping review limits itself to healthcare access research using the Levesque framework, it may simply be that experts and researchers in these countries are using other healthcare access frameworks. The results of this scoping review identified some studies [17, 19, 21–23, 25] which were able to explore access with a limited number of respondents but in a more in-depth manner when targeting a specific minority group.

### Tools to explore and/or measure healthcare access

The scoping review was able to identify a number of qualitative tools to explore healthcare access and quantitative tools to measure access. Considering the difficulty in measuring the complex concept that is healthcare access, a lot of studies on access use healthcare utilization as an alternative measure of access [53]. However, Levesque frameworks very clearly do not equate access to healthcare as simply the use of healthcare [6]. As such, none of the identified studies in this scoping review used healthcare utilization as a measure of access. Instead of measuring access as a whole, each of the identified quantitative and mixed-method studies measured each dimension of access. There are some dimensions of access that can be easily and directly measured as can be seen in two studies which tested measures for the affordability, availability, and accommodation dimensions of access [47, 48]. However, approachability and acceptability dimensions are more difficult to measure directly. Some questions on the tools intended to measure these dimensions, measures the patient’s or health provider’s perception of approachability or acceptability of health services. Another useful finding from this scoping review was that secondary data can be used to measure access to healthcare and its dimensions. This finding makes it possible for shorter data collection or assessment tools, should it be found that there are already existing national or community surveys conducted that contains data on certain dimensions or abilities of access to healthcare.

The Levesque framework’s main advantage over other access frameworks is its conceptualization of dimensions of access from a health systems perspective and population abilities to access for each of these dimensions [6]; surprisingly, many of the studies only focused on the dimensions of access or only on the abilities of people to access healthcare. The studies that looked into both dimensions and abilities of access showed that they have gathered more information on access and are thus able to explore, assess, or identify more clearly barriers to healthcare access.

Lastly, it is very noticeable that some dimensions and abilities are more represented than others when looking at the collated unique questions on access from the different tools from the studies included in this scoping review. As an example, there were a number of questions on the ability to perceive, while there were only a few questions on approachability. This discrepancy shows that at the initial part of the access process on recognizing the need for healthcare [6], more emphasis is given in assessing the ability of clients and patients to realize and acknowledge their healthcare needs as a result of their health literacy, health beliefs, and trust or expectations. The equally important task to assess the health systems or health facilities’ approachability through outreach efforts to improve awareness on the existence of the facility and its services as well as efforts for transparency to improve patients’ trust in the health facility and its health services [19], unfortunately are not given much attention. Another example is that there were a lot of questions on appropriateness but there was a paucity of questions on its counterpart’s ability to engage. This disparity shows that studies assessing access at the point of care almost always include quality, adequacy, and coordination, and seldom, empowerment and patient adherence. Recognition of this potential for imbalance would be useful for future researchers conducting studies on access to healthcare to enable them to take efforts and ensure that all dimensions and abilities of access are explored or measured.

## Limitations of the study

Healthcare access is a very broad concept where its definition, dimensions, and influencing factors are still continually debated internationally. This study only focuses on the healthcare access assessment through the lens of Conceptual Framework for Access to Healthcare by Levesque et al. Thus, there is a possibility that aspects of access not covered within the framework will also not be taken into consideration in this study. Also, this study only looks into empirical studies; non-empirical assessments of healthcare access with or without reference to the Levesque framework are not included. Lastly, only a third of the eligible respondent authors were interviewed making generalization difficult.

## Conclusion

The Levesque Conceptual Framework of Access to Healthcare has been successfully used to explore, assess, and measure access to healthcare in local, regional, national, and international studies in both HIC and LMIC settings. The framework allows for a very comprehensive look into the process-based concept of healthcare access starting from the ability to perceive the need for care and approachability of these healthcare services until after the healthcare services have been given by a health provider and received by a client or patient. The framework nonetheless also allows for more in-depth exploration and/or measurement of only selected dimensions or abilities of access for researchers interested in specific segments of the dynamic process of healthcare access. In addition, the framework’s design in which each access dimension has a corresponding ability allows easy recognition that both health systems and population context should always be taken into consideration in the healthcare access process. Considering all these, the use of the Conceptual Framework of Access to Healthcare would allow researchers to comprehensively assess the complex and dynamic process of access both in the health systems and in population context. Lastly, it might be worth including time-related elements of access such as travel time among the dimensions or sub-dimensions of access in order to more comprehensively evaluate access to healthcare.

## Supplementary Information

**Additional file 1.**

**Additional file 2.**

**Additional file 3.**

**Additional file 4.**

## Data Availability

Datasets used and analyzed in this scoping review are available from the corresponding author on request.
