# Supplementary material for: Assessing healthcare access using the Levesque’s conceptual framework– a scoping review
Source: Int J Equity Health. 2021 May 7;20:116. doi: 10.1186/s12939-021-01416-3 (PMC8103766; doi:10.1186/s12939-021-01416-3)
Supplement: Supplementary file 2 — Additional file 2. [file 12939_2021_1416_MOESM2_ESM.docx]

**Additional file 2**

*Assessing Healthcare Access Using the Levesque’s Conceptual Framework– A scoping Review*

**INTERVIEW GUIDE**

**Name: __________________________________________________________________**

**Date: ___________________________ Time : _______________________________**

**Introductions:**

Good day Mr/Ms. ____________, Thank you for taking time out of your busy schedule to accommodate my request for an interview.

- Introduce yourself
- Explain **the study and the** **purpose of the Interview** (read from the study information sheet)
- Follow up for **interview consent form** (if not yet secured)
- As for **permission to record** the interview

Do you have any questions before we start the interview?

**Interview Proper**

1. What made you and your team choose the Levesque Framework among the many other Framework for healthcare access?

*Probe: Ease or difficulty to use. Familiarity or prior use. Perceived advantages/disadvantages of the framework*

1. Can you explain to me in detail how you used the framework for your study?
2. If the framework was use *a priori* in the study: How did you find developing your assessment tool or interview guide using the framework?
3. If the framework was used *posteriori* in the study to organize data: How did the data collected fit into the different dimension of access based on the Levesque framework?
4. What is your experience with the use of the Levesque Framework in your study?
5. Did you experience any challenges in the use of the framework?
6. How did you cope with those challenges?
7. If you will use the Levesque Framework again in another research on healthcare access, what would you do differently and why?
8. Is there anything else you wish to add?

**Closing**

*Before we end this interview, do you have any questions?*

Thank you for participating in this interview.
