# Supplementary material for: Assessing healthcare access using the Levesque’s conceptual framework– a scoping review
Source: Int J Equity Health. 2021 May 7;20:116. doi: 10.1186/s12939-021-01416-3 (PMC8103766; doi:10.1186/s12939-021-01416-3)
Supplement: Supplementary file 3 — Additional file 3. [file 12939_2021_1416_MOESM3_ESM.docx]

***Additional file 3: Summary of Characteristics of Identified Studies***

|  | **Main Author** | **Year** | **Type of Study** | **Number of Respondents** | **Study Design** | **Geographical Scope of the Study** | **Classification of the Country Setting** | **Type of Data Analysis** |
| --- | --- | --- | --- | --- | --- | --- | --- | --- |
| 1 | Abdelwahab, M. | 2017 | Qualitative Research | 9 | Exploratory Design | Local | HIC | Narrative analysis |
| 2 | Abduludin, D. M.A. | 2019 | Qualitative Research | 10 | Phenomenology | Local | LMIC | Thematic Analysis |
| 3 | Anstey Watkins, J. | 2019 | Qualitative Research | 60 | Cross-sectional study | Local | LMIC | Thematic Analysis |
| 4 | Bailie, J. | 2015 | Mixed methods | 1044 | ND | National | HIC | Framework analysis |
| 5 | Bezem, J. | 2017 | Quantitative Descriptive | 1978 | Cross-sectional study | National | HIC | Statistical Analysis |
| 6 | Celik, LDC | 2016 | Qualitative Research | 12 | Exploratory Design | National | LMIC | Thematic Analysis |
| 7 | Chuah, F.L.H | 2018 | Qualitative Research | 20 | ND | National | LMIC | Thematic Analysis |
| 8 | Corscadden, L. | 2018 | Quantitative Descriptive | 20045 | ND | International | HIC | Statistical Analysis |
| 9 | Corscadden, L. | 2019 | Quantitative Descriptive | 5248 | ND | National | HIC | Statistical Analysis |
| 10 | Corscadden, L. | 2017 | Quantitative Descriptive | 2200 | ND | International | HIC | Statistical Analysis |
| 11 | Corscadden, L. | 2018 | Quantitative Descriptive | 23982 | ND | International | HIC | Statistical Analysis |
| 12 | Doetsch, J. | 2017 | Qualitative Research | 13 | Case study | Local | HIC | Content analysis |
| 13 | Fathi Afshar, S. | 2019 | Qualitative Research | 37 | ND | Local | HIC | Inductive - Deductive Analysis |
| 14 | Fauk, N.K. | 2019 | Qualitative Research | 29 | ND | Local | LMIC | Framework analysis |
| 15 | Gomez, H. | 2020 | Qualitative Research | 6 | Grounded theory | Local | LMIC | Thematic Analysis |
| 16 | Gordon, T. | 2017 | Quantitative Descriptive | 27580 |  | National | LMIC | Statistical Analysis |
| 17 | Haggerty, J. | 2017 | Mixed methods | 750 | Sequential exploratory design | National | HIC | Item Response Theory Analysis |
| 18 | Haggerty, J. | 2015 | Mixed methods | 12 | Sequential exploratory design | National | HIC | Statistical Analysis |
| 19 | Henry, J. | 2020 | Qualitative Research | 105 | Phenomenology | Local | HIC | Content analysis |
| 20 | Matthews, A. | 2019 | Qualitative Research | 6 | ND | Regional | LMIC | Framework analysis |
| 21 | McDonald, J. | 2015 | Mixed methods | 69 | Case study | Regional | HIC | Framework analysis |
| 22 | Miteniece, E. | 2018 | Qualitative Research | 63 |  | Local | LMIC | Content analysis |
| 23 | Miteniece, E. | 2019 | Mixed methods | 372 | Sequential exploratory design | National | HIC | Thematic Analysis |
| 24 | Packness, A. | 2019 | Quantitative Descriptive | 744 | Cross-sectional study | Local | HIC | Statistical Analysis |
| 25 | Richard, L. | 2019 | Mixed methods | 740 | Survey | International | HIC | Framework analysis |
| 26 | Roberge, P. | 2014 | Quantitative Descriptive |  | Cohort study | Local | HIC | Statistical Analysis |
| 27 | Russell, G. | 2016 | Mixed methods | 25 | Convergent design | International | HIC | Thematic Analysis |
| 28 | Tam, W.J. | 2017 | Qualitative Research | 30 | ND | Local | HIC | Thematic Analysis |
| 29 | Vandan, N. | 2019 | Qualitative Research | 16 | ND | Local | HIC | Framework analysis |
| 30 | Viveiros, C. J. | 2018 | Qualitative Research | 6 | Qualitative description | Local | HIC | Thematic Analysis |
| 31 | Ward, B. | 2018 | Mixed methods | 750 | Case study | National | HIC | Thematic Analysis |
