# Supplementary material for: Assessing healthcare access using the Levesque’s conceptual framework– a scoping review
Source: Int J Equity Health. 2021 May 7;20:116. doi: 10.1186/s12939-021-01416-3 (PMC8103766; doi:10.1186/s12939-021-01416-3)
Supplement: Supplementary file 4 — Additional file 4. [file 12939_2021_1416_MOESM4_ESM.docx]

***Additional file 4: List of Extracted Unique Questions on Access to Healthcare***

|  | **Question** |  | **Scale (N)** | **Scale (Description)** | **Qualitative Vs Quantitative** | **Dimension / Ability Classification** | **Sub-dimension / Sub-ability** | **Question for**  **Health Providers Vs Recipients** |
| --- | --- | --- | --- | --- | --- | --- | --- | --- |
| 1 | How informed do you think you are about care in maternal period and about maternal care services? | Maternal and Child Care | N/A | N/A | Qualitative | Approachability | Information Screening | Recipients |
| 2 | What do you think about the dissemination of information about HIV related health services in your community? | Infectious Diseases / HIV/AIDS Health Services | N/A | N / A | Qualitative | Approachability | Information Screening | Recipients |
| 3. | Do you feel like you missed any important information during the maternal period? | Maternal and Child Care |  | Yes | Quantitative | Approachability | Transparency | Recipients |
|  |  |  |  | No |  |  |  |  |
| 4. | How would you rate the overall quality of medical care in your country/community? | General Health Services / Primary Health Care | 1 | Excellent | Quantitative | Approachability | Information Screening | Recipients |
|  |  |  | 2 | Very Good |  |  |  |  |
|  |  |  | 3 | Good |  |  |  |  |
|  |  |  | 4 | Fair |  |  |  |  |
|  |  |  | 5 | Poor |  |  |  |  |
|  |  |  | 8 | Not sure |  |  |  |  |
|  |  |  | 9 | Decline to answer |  |  |  |  |
| 5. | Is there one doctor you usually go to for your medical care? | General Health Services / Primary Health Care | 1 | Yes, I have a regular Doctor | Quantitative | Approachability | Information Screening | Recipients |
|  |  |  | 2 | Yes, but have more than 1 regular doctor/GP |  |  |  |  |
|  |  |  | 3 | No |  |  |  |  |
|  |  |  | 8 | Not Sure |  |  |  |  |
|  |  |  | 9 | Decline to answer |  |  |  |  |
| 6. | Is there a health center, or clinic you usually, go to for most of your care? | General Health Services / Primary Health Care | 1 | Yes | Quantitative | Approachability | Information Screening | Recipients |
|  |  |  | 2 | No |  |  |  |  |
|  |  |  | 8 | Not Sure |  |  |  |  |
|  |  |  | 9 | Decline to answer |  |  |  |  |
| 7. | Which of the following statements comes closest to expressing your overall view of the health‐care system in your country? | General Health Services / Primary Health Care | 1 | On the whole, the system works pretty well and only minor changes are necessary to make it work better. | Quantitative | Approachability | Transparency | Recipients |
|  |  |  | 2 | There are some good things in our health care system, but fundamental changes are needed to make it work better |  |  |  |  |
|  |  |  | 3 | Our health care system has so much wrong with it that we need to completely rebuild it. |  |  |  |  |
|  |  |  | 8 | Not Sure |  |  |  |  |
|  |  |  | 9 | Decline to answer |  |  |  |  |
| 8. | (Reflect on language, cultural, and social factors determine refugees’ ability to obtain and accept care)  To what extent do you experience that language, culture, and religion influence the care you provide? | General Health Services / Primary Health Care | N/A | N / A | Qualitative | Acceptability | Culture | Health Providers |
| 9. | What are some of the cultural barriers you’ve faced in accessing healthcare? | General Health Services / Primary Health Care | N/A | N / A | Qualitative | Acceptability | Culture | Recipients |
| 10. | How do you think healthcare providers can be culturally more sensitive to you and your needs here in their delivery of healthcare? | General Health Services / Primary Health Care | N/A | N / A | Qualitative | Acceptability | Culture | Recipients |
| 11. | How do you feel about the services on HIV treatment that is provided by doctors and nurses? | Infectious Diseases / HIV/AIDS Health Services | N/A | N / A | Qualitative | Acceptability | Professional values | Recipients |
| 12. | How do you feel about the attitude and behaviours of health workers when providing HIV/AIDS services? | Infectious Diseases / HIV/AIDS Health Services | N/A | N / A | Qualitative | Acceptability | Professional values | Recipients |
| 13. | Do you think it is important to receive maternal healthcare services during antenatal period, childbirth and postnatal period? | Maternal and Child Care | N/A | N/A | Qualitative | Acceptability | Personal and social values | Recipients |
| 14. | Why do you think it is important to receive  maternal healthcare services during antenatal period, childbirth and postnatal period? | Maternal and Child Care | N/A | N/A | Qualitative | Acceptability | Personal and social values | Recipients |
| 15. | Do  you feel that you have had as much support from health professionals as you needed to help you manage your health problems? | General Health Services / Primary Health Care | 1 | Yes, definitely | Quantitative | Acceptability | Caregiver support | Recipients |
|  |  |  | 2 | Yes, to some extent |  |  |  |  |
|  |  |  | 3 | No |  |  |  |  |
|  |  |  | 8 | Not Sure |  |  |  |  |
|  |  |  | 9 | Decline to answer |  |  |  |  |
| 16. | When you contact your regular doctor's office with a medical question during regular practice hours, how often do you get an answer that same day? | General Health Services / Primary Health Care | 1 | Always | Quantitative | Acceptability | Appointments mechanisms | Recipients |
|  |  |  | 2 | Often |  |  |  |  |
|  |  |  | 3 | Sometimes |  |  |  |  |
|  |  |  | 4 | Rarely or never |  |  |  |  |
|  |  |  | 5 | Never tried to contact |  |  |  |  |
| 17. | What are the procedures to access HIV/AIDS-related health services in your community? | Infectious Diseases / HIV/AIDS Health Services | N/A | N / A | Qualitative | Availability/  Accommodation | Appointments mechanisms | Recipients |
| 18. | Did you experience any maternal care services not being available in the area you live (either not existent or shortage of availability) during antenatal, delivery or post-natal period? | Maternal and Child Care | N/A | N / A | Qualitative | Availability/  Accommodation | Accommodation | BOTH |
| 19. | Did you experience any issues to access maternal care services during antenatal, delivery or post-natal period in terms of distance or time? | Maternal and Child Care | N/A | N / A | Qualitative | Availability/  Accommodation | Geographic location | Recipients |
| 20 | In your opinion, what are the main barriers to seek for the necessary maternal healthcare service in terms of space and time? | Maternal and Child Care | N/A | N / A | Qualitative | Availability/  Accommodation | Geographic location | BOTH |
| 21. | What kind of health services are refugee children and unaccompanied minors offered in your country? | General Health Services / Primary Health Care | N/A | N/A | Qualitative | Availability/  Accommodation | Accommodation | Health Providers |
| 22. | How do you think the current situation of maternal care availability in your community influences health outcomes of the mothers and their babies? | Maternal and Child Care | N/A | N/A | Qualitative | Availability/  Accommodation | Accommodation | Health Providers |
| 23. | What types of HIV/AIDS-related health services are available in your community? | Infectious Diseases / HIV/AIDS Health Services | N/A | N/A | Qualitative | Availability/  Accommodation | Accommodation | Recipients |
| 24. | Are HIV/AIDs services available in the nearest health facilities in your community? | Infectious Diseases / HIV/AIDS Health Services | N/A | N/A | Qualitative | Availability/  Accommodation | Geographic location | Recipients |
| 25. | Did your health facility ever experienced, a shortage of medical staff, which in your opinion hindered the deliver adequate services to patients? | General Health Services / Primary Health Care | N/A | Yes / No | Qualitative | Availability/  Accommodation | Accommodation | Health Providers |
| 26. | Do you think there is enough available of healthcare services (hospitals, specialized physicians, procedures, etc) in your community? | General Health Services / Primary Health Care | N/A | N / A | Qualitative | Availability/  Accommodation |  | Recipients |
| 27. | How available are maternal care services for antenatal period, childbirth or postnatal period in your community? | Maternal and Child Care |  | Not available | Quantitative | Availability/  Accommodation | Accommodation | Health Providers |
|  |  |  |  | Not always available |  |  |  |  |
|  |  |  |  | Available, but not good quality |  |  |  |  |
|  |  |  |  | Available, with good quality |  |  |  |  |
| 28. | Is the clinic close to your home? | General Health Services / Primary Health Care | 1 | Very far | Quantitative | Availability/  Accommodation | Geographic location | Recipients |
|  |  |  | Scale for 2, 3 and 4 not provided in the article. | |  |  |  |  |
|  |  |  | 5 | Very Close |  |  |  |  |
| 29. | How long does it take you to get to your clinic? | General Health Services / Primary Health Care | 1 | More than one hour | Quantitative | Availability/  Accommodation | Geographic location | Recipients |
|  |  |  | Scale for 2, 3 and 4 not provided in the article. | |  |  |  |  |
|  |  |  | 5 | Less than 5 min |  |  |  |  |
| 30. | For your routine or non-urgent health-care needs, how easy is it to travel to the hospital/clinic? | General Health Services / Primary Health Care | 1 | Not at all easy | Quantitative | Availability/  Accommodation | Geographic location | Recipients |
|  |  |  | Scale for 2, 3 and 4 not provided in the article. | |  |  |  |  |
|  |  |  | 5 | Very easy |  |  |  |  |
| 31. | When you need immediate care, how easy is it to get to the hospital/clinic? | General Health Services / Primary Health Care | 1 | Not at all easy | Quantitative | Availability/  Accommodation | Geographic location | Recipients |
|  |  |  | Scale for 2, 3 and 4 not provided in the article. | |  |  |  |  |
|  |  |  | 5 | Very easy |  |  |  |  |
| 32. | What phrase best describes the number of healthcare clinics present in your neighbourhood? | General Health Services / Primary Health Care | 1 | None for miles | Quantitative | Availability/  Accommodation | Geographic location | Recipients |
|  |  |  | Scale for 2, 3 and 4 not provided in the article. | |  |  |  |  |
|  |  |  | 5 | Many clinics nearby |  |  |  |  |
| 33. | After you were advised to see or decided to see a specialist, how long did you have to wait for an appointment? | Specialty Care | 1 | Days | Quantitative | Availability/  Accommodation | Appointments mechanisms | Recipients |
|  |  |  | 2 | Weeks |  |  |  |  |
|  |  |  | 3 | Months |  |  |  |  |
|  |  |  | 4 | Years |  |  |  |  |
|  |  |  | 5 | Never tried |  |  |  |  |
|  |  |  | 6 | No waiting period |  |  |  |  |
|  |  |  | 8 | Not Sure |  |  |  |  |
|  |  |  | 9 | Decline to answer |  |  |  |  |
| 34. | Last time you were sick or needed medical attention, how quickly could you get an appointment to see a doctor or a nurse? | General Health Services / Primary Health Care | 1 | On the same day | Quantitative | Availability/  Accommodation | Appointments mechanisms | Recipients |
|  |  |  | 2 | The next day |  |  |  |  |
|  |  |  | 3 | In 2 to 5 days |  |  |  |  |
|  |  |  | 4 | In 6 to 7 days |  |  |  |  |
|  |  |  | 5 | In 8 to 14 days |  |  |  |  |
|  |  |  | 6 | After more than two weeks |  |  |  |  |
|  |  |  | 7 | never able to get an appointment |  |  |  |  |
|  |  |  | 0 | Did not need to make an  appointment to see doctor or nurse |  |  |  |  |
|  |  |  | 8 | Not Sure |  |  |  |  |
|  |  |  | 9 | Decline to answer |  |  |  |  |
| 35. | How easy or difficult is it to get medical care in the evenings, on weekends, or holidays without going to the hospital emergency department? | General Health Services / Primary Health Care | 1 | Very Easy | Qualitative | Availability/  Accommodation | Appointments mechanisms | Recipients |
|  |  |  | 2 | Somewhat easy |  |  |  |  |
|  |  |  | 3 | Somewhat difficult |  |  |  |  |
|  |  |  | 4 | Very Difficult |  |  |  |  |
|  |  |  | 5 | Never needed care in the evenings, weekends or holidays |  |  |  |  |
|  |  |  | 8 | Not Sure |  |  |  |  |
|  |  |  | 9 | Decline to answer |  |  |  |  |
| 36. | How do you rate the usual waiting time for an appointment with your doctor? | General Health Services / Primary Health Care | 1 | Very Poor | Quantitative | Availability/  Accommodation | Appointments mechanisms | Recipients |
|  |  |  | Scale for 2, 3 and 4 not provided in the article. | |  |  |  |  |
|  |  |  | 5 | Very Good |  |  |  |  |
| 37. | At your clinic, if you need to be seen quickly, how easy would it be to be seen sooner than the usual appointment time? | General Health Services / Primary Health Care | 1 | not at all easy | Quantitative | Availability/  Accommodation | Appointments mechanisms | Recipients |
|  |  |  | Scale for 2, 3 and 4 not provided in the article. | |  |  |  |  |
|  |  |  | 5 | very easy |  |  |  |  |
| 38. | In your experience, to what extent does the patients have to pay extra for transport, medications or additional medical assistance? | General Health Services / Primary Health Care | N/A | N / A | Qualitative | Affordability | Direct costs | Health Providers |
| 39. | In your experience, to what extent are your patients aware of their health insurance and their deductible to use the necessary care? | General Health Services / Primary Health Care | N/A | N / A | Qualitative | Affordability | Direct costs | Health Providers |
| 40. | Do you think that the payments for maternal care serves as a barrier to access or use certain services? | Maternal and Child Care | N/A | N / A | Qualitative | Affordability | Direct costs | BOTH |
| 41. | Do you think there is an inherent difference in quality between maternal care services provided between public and private health providers in your community? | Maternal and Child Care | N/A | N / A | Qualitative | Affordability | Direct costs | BOTH |
| 42. | Would your country's childbearing women be expected to make unofficial payments [sometimes described as 'under-the-table' payments] to doctors for their services (in addition to any official co-payment of appointment fees)? | General Health Services / Primary Health Care | N/A | Yes / No | Qualitative | Affordability | Indirect costs | BOTH |
| 43. | What is the reason for unofficial payments [sometimes described as 'under-the-table' payments] to doctors or healthcare workers in your community? | Maternal and Child Care | N/A | N / A | Qualitative | Affordability | Indirect costs | BOTH |
| 4.4. | How do you the think the current cost of maternal care services, influences care seeking behaviour and health outcomes of women and their babies in your community? | Maternal and Child Care | N/A | N / A | Qualitative | Affordability | Direct costs | BOTH |
| 45. | Where do you plan to receive your maternal care services need, in the public or private sector? | Maternal and Child Care | N/A | 1= Public 2= Private 3= Mix of Public and Private | Qualitative | Affordability | Direct costs | Recipients |
| 46. | Are HIV/AIDS-related services provided for free in your community? | Infectious Diseases / HIV/AIDS Health Services | N/A | N / A | Qualitative | Affordability | Direct costs | Recipients |
| 47. | What do you think about the costs of HIV/AIDS-related services? | Infectious Diseases / HIV/AIDS Health Services | N/A | N / A | Qualitative | Affordability | Direct costs | Recipients |
| 48. | What maternal and child care services did you have to pay for? | Maternal and Child Care | N/A | N / A | Qualitative | Affordability | Direct costs | Recipients |
| 49. | How much does pregnant women have to pay for maternal care services in your community? | Maternal and Child Care |  | Give Exact Amount | Quantitative | Affordability | Direct costs | BOTH |
| 50. | Are there times when you don't take drugs prescribed by a doctor because of the costs? | General Health Services / Primary Health Care | 0 | Never, rarely | Quantitative | Affordability | Direct costs | Recipients |
|  |  |  | 1 | Sometimes, Often, Very often |  |  |  |  |
| 51. | Are there times when you don't take laboratory tests or exams because of the costs? | General Health Services / Primary Health Care | 0 | Never, rarely | Quantitative | Affordability | Direct costs | Recipients |
|  |  |  | 1 | Sometimes, Often, Very often |  |  |  |  |
| 52. | Have there been instances when you decided not to get services prescribed by a doctor but not covered by health insurance because of the costs it entails? | General Health Services / Primary Health Care | 0 | Never, rarely | Quantitative | Affordability | Direct costs | Recipients |
|  |  |  | 1 | Sometimes, Often, Very often |  |  |  |  |
| 53. | Are there times when you find it difficult to get health care services because of the loss of income it involves? | General Health Services / Primary Health Care | 0 | Never, rarely | Quantitative | Affordability | Opportunity costs | Recipients |
|  |  |  | 1 | Sometimes, Often, Very often |  |  |  |  |
| 54. | Are there times when you find it difficult to get health care services because of the additional costs it involves (babysitting, parking, etc.)? | General Health Services / Primary Health Care | 0 | Never, rarely | Quantitative | Affordability | Indirect costs | Recipients |
|  |  |  | 1 | Sometimes, Often, Very often |  |  |  |  |
| 55. | In the past 12 months, were there times when you had serious problems paying or were unable to pay your medical bills? | General Health Services / Primary Health Care | 1 | Yes | Quantitative | Affordability | Direct costs | Recipients |
|  |  |  | 2 | No |  |  |  |  |
|  |  |  | 8 | Not Sure |  |  |  |  |
|  |  |  | 9 | Decline to answer |  |  |  |  |
| 56. | In the past 12 months, were there times when you had dental problems but did not seek consult due to the cost? | Oral and Dental Health | 1 | Yes | Quantitative | Affordability | Direct costs | Recipients |
|  |  |  | 2 | No |  |  |  |  |
|  |  |  | 8 | Not Sure |  |  |  |  |
|  |  |  | 9 | Decline to answer |  |  |  |  |
| 57. | In the past 12 months, were there times when you spent a lot of time on paperwork or disputes related to medical bills? | General Health Services / Primary Health Care | 1 | Yes | Quantitative | Affordability | Direct costs | Recipients |
|  |  |  | 2 | No |  |  |  |  |
|  |  |  | 8 | Not Sure |  |  |  |  |
|  |  |  | 9 | Decline to answer |  |  |  |  |
| 58. | In the past 12 months, were there times when your insurance denied payment for your medical care or did not pay as much as you expected? | General Health Services / Primary Health Care | 1 | Yes | Quantitative | Affordability | Direct costs | Recipients |
|  |  |  | 2 | No |  |  |  |  |
|  |  |  | 8 | Not Sure |  |  |  |  |
|  |  |  | 9 | Decline to answer |  |  |  |  |
| 59. | Do you feel you get support from the HIV Treatment Center health workers when you access services? | Infectious Diseases / HIV/AIDS Health Services | N/A | N / A | Qualitative | Appropriateness | Interpersonal Quality | Recipients |
| 60. | Do you think that your patients understand you sufficiently during your consultation? | General Health Services / Primary Health Care | N/A | Yes / No | Qualitative | Appropriateness | Adequacy | Health Providers |
| 61.. | How do you ensure that your patients/clients understand your instructions? | General Health Services / Primary Health Care | N/A | N / A | Qualitative | Appropriateness | Adequacy | Health Providers |
| 62. | What do you think are the reason why patients have problems understanding doctors or health workers instructions? | General Health Services / Primary Health Care | N/A | N / A | Qualitative | Appropriateness | Adequacy | Health Providers |
| 63. | In your opinion, is the quality of maternal care women receive during antenatal period, childbirth and postnatal period in your community irrespective to their socio-economic status or health condition? | Maternal and Child Care | N/A | N / A | Qualitative | Appropriateness | Technical Quality | BOTH |
| 64. | Do you think the HIV/AIDS-related health services available in your community are appropriate for the ones that you need? | Infectious Diseases / HIV/AIDS Health Services | N/A | N / A | Qualitative | Appropriateness | Technical Quality | Recipients |
| 65. | Do you think healthcare services are delivered in a good way by qualified health service providers in your community? | General Health Services / Primary Health Care | N/A | N / A | Qualitative | Appropriateness | Technical Quality | Recipients |
| 66. | Were you satisfied with attitude of maternal care providers and with the way they communicated with you during your last visit? | Maternal and Child Care | N/A | N / A | Qualitative | Appropriateness | Interpersonal Quality | Recipients |
| 67. | Do you receive a timely response from School Health Services (SHS) to your questions about children with specific needs? | School Health Services | 1 | Never | Quantitative | Appropriateness | Adequacy | Health Providers |
|  |  |  | 2 | Sometimes |  |  |  |  |
|  |  |  | 3 | Often |  |  |  |  |
|  |  |  | 4 | Always |  |  |  |  |
|  |  |  | 5 | Not Applicable |  |  |  |  |
| 68. | Are you satisfied with the number of contacts with School Health Services professionals? | School Health Services |  | Yes | Quantitative | Appropriateness | Adequacy | Health Providers |
|  |  |  |  | No |  |  |  |  |
| 69. | Do you receive useful feedback from School Health Services on your questions about children with specific needs? | School Health Services | 1 | Never | Quantitative | Appropriateness | Coordination and continuity | Health Providers |
|  |  |  | 2 | Sometimes |  |  |  |  |
|  |  |  | 3 | Often |  |  |  |  |
|  |  |  | 4 | Always |  |  |  |  |
|  |  |  | 5 | Not Applicable |  |  |  |  |
| 70. | Do you receive sufficient feedback from School Health Services after the health assessments? | School Health Services | 1 | Never | Quantitative | Appropriateness | Coordination and continuity | Health Providers |
|  |  |  | 2 | Sometimes |  |  |  |  |
|  |  |  | 3 | Often |  |  |  |  |
|  |  |  | 4 | Always |  |  |  |  |
|  |  |  | 5 | Not Applicable |  |  |  |  |
| 71. | To what extent do you agree or disagree with the statement: I contact School Health Services when I have concerns about a pupils’ health? | School Health Services | 1 | Strongly Disagree | Quantitative | Appropriateness | Coordination and continuity | Health Providers |
|  |  |  | 2 | Disagree |  |  |  |  |
|  |  |  | 3 | Neutral |  |  |  |  |
|  |  |  | 4 | Agree |  |  |  |  |
|  |  |  | 5 | Strongly agree |  |  |  |  |
|  |  |  | 6 | No Opinion / Don’t Know |  |  |  |  |
| 72. | To what extent do you agree or disagree with the statement: I contact School Health Services when I have concerns about a pupils’ psychosocial development? | School Health Services | 1 | Strongly Disagree | Quantitative | Appropriateness | Coordination and continuity | Health Providers |
|  |  |  | 2 | Disagree |  |  |  |  |
|  |  |  | 3 | Neutral |  |  |  |  |
|  |  |  | 4 | Agree |  |  |  |  |
|  |  |  | 5 | Strongly agree |  |  |  |  |
|  |  |  | 6 | No Opinion / Don’t Know |  |  |  |  |
| 73. | Overall, how would you rate the medical care that you have received in the past 12 months from your regular GP's practice? | General Health Services / Primary Health Care | 1 | Excellent | Quantitative | Appropriateness | Technical Quality | Recipients |
|  |  |  | 2 | Very Good |  |  |  |  |
|  |  |  | 3 | Good |  |  |  |  |
|  |  |  | 4 | Fair |  |  |  |  |
|  |  |  | 5 | Poor |  |  |  |  |
|  |  |  | 7 | No health services received in the last 12 months |  |  |  |  |
|  |  |  | 8 | Not Sure |  |  |  |  |
|  |  |  | 9 | Decline to answer |  |  |  |  |
| 74. | During your hospital stay, how often did doctors treat you with courtesy and respect? | General Health Services / Primary Health Care | 1 | Always | Quantitative | Appropriateness | Interpersonal Quality | Recipients |
|  |  |  | 2 | Sometimes |  |  |  |  |
|  |  |  | 3 | Rarely |  |  |  |  |
|  |  |  | 4 | Never |  |  |  |  |
|  |  |  | 8 | Not Sure |  |  |  |  |
|  |  |  | 9 | Decline to answer |  |  |  |  |
| 75. | During this hospital stay, how often did nurses treat you with courtesy and respect? | General Health Services / Primary Health Care | 1 | Always  Sometimes  Rarely  Never  Not Sure  Decline to answer | Quantitative | Appropriateness | Interpersonal Quality | Recipients |
|  |  |  | 2 |  |  |  |  |  |
|  |  |  | 3 |  |  |  |  |  |
|  |  |  | 4 |  |  |  |  |  |
|  |  |  | 8 |  |  |  |  |  |
|  |  |  | 9 |  |  |  |  |  |
| 76. | To what extent do you agree or disagree with the statement: School Health Services School Health Services provides an important contribution in detecting problems? | School Health Services | 1 | Stronly Disagree | Quantitative | Appropriateness | Trust and expectations | Health Providers |
|  |  |  | 2 | Disagree |  |  |  |  |
|  |  |  | 3 | Neutral |  |  |  |  |
|  |  |  | 4 | Agree |  |  |  |  |
|  |  |  | 5 | Strongly agree |  |  |  |  |
|  |  |  | 6 | No Opinion /Don’t Know |  |  |  |  |
| 77. | To what extent do you agree or disagree with the statement: School Health Services ensures children with specific needs are referred to proper care in time? | School Health Services | 1 | Stronly Disagree | Quantitative | Appropriateness | Coordination and continuity | Health Providers |
|  |  |  | 2 | Disagree |  |  |  |  |
|  |  |  | 3 | Neutral |  |  |  |  |
|  |  |  | 4 | Agree |  |  |  |  |
|  |  |  | 5 | Strongly agree |  |  |  |  |
|  |  |  | 6 | No Opinion /Don’t Know |  |  |  |  |
| 78. | To what extent do you agree or disagree with the statement: School Health Services gives sufficient attention to children with specific needs? | School Health Services | 1 | Strongly Disagree | Quantitative | Appropriateness | Technical Quality and Adequacy | Health Providers |
|  |  |  | 2 | Disagree |  |  |  |  |
|  |  |  | 3 | Neutral |  |  |  |  |
|  |  |  | 4 | Agree |  |  |  |  |
|  |  |  | 5 | Strongly agree |  |  |  |  |
|  |  |  | 6 | No Opinion /Don’t Know |  |  |  |  |
| 79. | During the past year, when you received mental health care, has any health‐care professional given you a written plan to help you manage your own care? | Mental Health | 1 | Yes | Quantitative | Appropriateness | Information | Recipients |
|  |  |  | 2 | No |  |  |  |  |
|  |  |  | 8 | Not Sure |  |  |  |  |
|  |  |  | 9 | Decline to answer |  |  |  |  |
| 80. | Thinking about the past two years, when receiving care for a medical problem, was there ever a time when you received conflicting information from different doctors or health‐care professionals? | General Health Services / Primary Health Care | 1 | Yes | Quantitative | Appropriateness | Coordination and continuity | Recipients |
|  |  |  | 2 | No |  |  |  |  |
|  |  |  | 8 | Not Sure |  |  |  |  |
|  |  |  | 9 | Decline to answer |  |  |  |  |
| 81. | How often does your regular doctor or someone in your doctor's practice/GP's practice help coordinate or arrange the care you receive from other doctors and places? | General Health Services / Primary Health Care | 1 | Always | Quantitative | Appropriateness | Coordination and continuity | Recipients |
|  |  |  | 2 | Often |  |  |  |  |
|  |  |  | 3 | Sometimes |  |  |  |  |
|  |  |  | 4 | Rarely or never |  |  |  |  |
|  |  |  | 5 | Did not need to see any other doctors/health professionals or did not need any coordination |  |  |  |  |
|  |  |  | 8 | Not Sure |  |  |  |  |
|  |  |  | 9 | Decline to answer |  |  |  |  |
| 82. | In the past two years, have you experienced the following: After you saw the specialist, your regular GP did not seem informed and up‐to‐date about the care you got? | General Health Services / Primary Health Care | 1 | Yes | Quantitative | Appropriateness | Coordination and continuity | Recipients |
|  |  |  | 2 | No |  |  |  |  |
|  |  |  | 8 | Not Sure |  |  |  |  |
|  |  |  | 9 | Decline to answer |  |  |  |  |
| 83. | In the past two years, have you experienced the following: The specialist did not have basic medical information or test results from your regular doctor about the reason for your visit? | General Health Services / Primary Health Care | 1 | Yes | Quantitative | Appropriateness | Coordination and continuity | Recipients |
|  |  |  | 2 | No |  |  |  |  |
|  |  |  | 8 | Not Sure |  |  |  |  |
|  |  |  | 9 | Decline to answer |  |  |  |  |
| 84. | Thinking about the past two years, when receiving care for a medical problem, was there ever a time when test results or medical records were not available at the time of your scheduled medical care appointment? | General Health Services / Primary Health Care | 1 | Yes | Quantitative | Appropriateness | Coordination and continuity | Recipients |
|  |  |  | 2 | No |  |  |  |  |
|  |  |  | 8 | Not Sure |  |  |  |  |
|  |  |  | 9 | Decline to answer |  |  |  |  |
| 85. | When you left the hospital, did the hospital make arrangements or make sure you had follow‐up care with a doctor or other health‐care professional? | General Health Services / Primary Health Care | 1 | Yes | Quantitative | Appropriateness | Coordination and continuity | Recipients |
|  |  |  | 2 | No |  |  |  |  |
|  |  |  | 8 | Not Sure |  |  |  |  |
|  |  |  | 9 | Decline to answer |  |  |  |  |
| 86. | When you left the hospital, did someone discuss with you the purpose of taking each of your medications? | General Health Services / Primary Health Care | 1 | Yes | Quantitative | Appropriateness | Coordination and continuity | Recipients |
|  |  |  | 2 | No |  |  |  |  |
|  |  |  | 8 | Not Sure |  |  |  |  |
|  |  |  | 9 | Decline to answer |  |  |  |  |
| 87. | When you left the hospital, did you receive written information on what to do when you returned home and what symptoms to watch for? | General Health Services / Primary Health Care | 1 | Yes | Quantitative | Appropriateness | Coordination and continuity | Recipients |
|  |  |  | 2 | No |  |  |  |  |
|  |  |  | 8 | Not Sure |  |  |  |  |
|  |  |  | 9 | Decline to answer |  |  |  |  |
| 88. | When you need care or treatment, how often does your regular doctor or medical/your GP or medical/the doctor or medical staff you see explain things in a way that is easy to understand? | General Health Services / Primary Health Care | 1 | Always | Quantitative | Appropriateness | Adequacy | Recipients |
|  |  |  | 2 | Often |  |  |  |  |
|  |  |  | 3 | Sometimes |  |  |  |  |
|  |  |  | 4 | Rarely or never |  |  |  |  |
|  |  |  | 5 | Not Applicable |  |  |  |  |
|  |  |  | 8 | Not Sure |  |  |  |  |
|  |  |  | 9 | Decline to answer |  |  |  |  |
| 89. | When you need care or treatment, how often does your regular GP or medical staff you see spend enough time with you? | General Health Services / Primary Health Care | 1 | Always | Quantitative | Appropriateness | Adequacy | Recipients |
|  |  |  | 2 | Often |  |  |  |  |
|  |  |  | 3 | Sometimes |  |  |  |  |
|  |  |  | 4 | Rarely or never |  |  |  |  |
|  |  |  | 5 | Not Applicable |  |  |  |  |
|  |  |  | 8 | Not Sure |  |  |  |  |
|  |  |  | 9 | Decline to answer |  |  |  |  |
| 90. | During your last visit to a health facility, were you satisfied with the skills of healthcare professionals and conditions at facility? | General Health Services / Primary Health Care | 1 | Yes | Quantitative | Appropriateness | Technical Quality | Recipients |
|  |  |  | 2 | No |  |  |  |  |
|  |  |  | 8 | Not Sure |  |  |  |  |
|  |  |  | 9 | Decline to answer |  |  |  |  |
| 91. | Overall, were you satisfied with the services received during maternal period? | Maternal and Child Care |  | Yes | Quantitative | Appropriateness | Adequacy | Recipients |
|  |  |  |  | No |  |  |  |  |
| 92. | Where do pregnant women get information about maternal health? | Maternal and Child Care | N/A | N / A | Qualitative | To Perceive | Health literacy | Recipients |
| 93. | What do you know about Voluntary Termination of Pregnancy? | Maternal and Child Care | N/A | N / A | Qualitative | To Perceive | Health literacy | Recipients |
| 94. | What do you know about family planning methods? | Maternal and Child Care | N/A | N / A | Qualitative | To Perceive | Health literacy | Recipients |
| 95. | What do you know about Zika Virus and its effects on pregnancy? | Infectious Diseases / HIV/AIDS Health Services | N/A | N / A | Qualitative | To Perceive | Health literacy | Recipients |
| 96. | Do you think that the limited quality of maternal healthcare services is a barrier to seek for the care (e.g. poor attitude, conditions in healthcare unit, treatment itself)? Why? | Maternal and Child Care | N/A | N / A | Qualitative | To Perceive | Trust and expectations | BOTH |
| 97. | Do you trust in the healthcare system? | General Health Services / Primary Health Care | N/A | N / A | Qualitative | To Perceive | Trust and expectations | Recipients |
| 98. | Do you trust information or treatments provided by physicians/nurses regarding Zika and its effects on pregnancy? Explain | Infectious Diseases / HIV/AIDS Health Services | N/A | N / A | Qualitative | To Perceive | Trust and expectations | Recipients |
| 99. | What do refugee parents and Unaccompanied Minor Aliens know about the health system and their right to healthcare?’ | General Health Services / Primary Health Care | N/A | N/A | Qualitative | To Perceive | Health literacy | Health Providers |
| 100. | Do you consider yourself healthy? Why? | General Health Services / Primary Health Care | N/A | Yes / No | Qualitative | To Perceive | Health beliefs | Recipients |
| 101. | Do you have any chronic problem? | General Health Services / Primary Health Care | N/A | Yes / No | Qualitative | To Perceive | Health literacy | Recipients |
| 102. | What kind of health problems you are suffering from? | General Health Services / Primary Health Care | N/A | N / A | Qualitative | To Perceive | Health literacy | Recipients |
| 103. | Do you perceive a need to receive maternal healthcare services during all the three phases – pre/postnatal and delivery? Why? | Maternal and Child Care | N/A | N / A | Qualitative | To Perceive | Health beliefs | Health Providers |
| 104. | Could you tell whether there are any maternal care services that you think are not necessary/important? | Maternal and Child Care | N/A | N / A | Qualitative | To Perceive | Health beliefs | Health Providers |
| 105. | Do you know of any reason why pregnant women chooses not to seek maternal health services? | Maternal and Child Care | N/A | N / A | Qualitative | To Perceive | Health beliefs | Health Providers |
| 106. | Did you feel like you were missing information on the use of maternal services? | Maternal and Child Care | N/A | N / A | Qualitative | To Perceive | Health literacy | Health Providers |
| 107. | What factors do you think influences how women perceive the need for maternal healthcare services in your community | Maternal and Child Care | N/A | N / A | Qualitative | To Perceive | Health beliefs | Health Providers |
| 108. | In your experience, how does educational attainment and health literacy about maternal care services affects a person's use of maternal and child care services? | Maternal and Child Care | N/A | N / A | Qualitative | To Perceive | Health literacy | Health Providers |
| 109. | How do you think the current level of maternal care acceptance and general health literacy in your community influences the health outcomes of women and their babies? | Maternal and Child Care | N/A | N / A | Qualitative | To Perceive | Trust and expectations | Health Providers |
| 110. | How do you know about the availability of HIV/AIDS-related health services? | Infectious Diseases / HIV/AIDS Health Services | N/A | N / A | Qualitative | To Perceive | Health literacy | Recipients |
| 111. | How did you learn about HIV/AIDS? | Infectious Diseases / HIV/AIDS Health Services | N/A | N / A | Qualitative | To Perceive | Health literacy | Recipients |
| 112. | How confident are you that you can control and manage your health problems? | General Health Services / Primary Health Care | 1 | Very confident | Quantitative | To Perceive | Health beliefs | Recipients |
|  |  |  | 2 | Confident |  |  |  |  |
|  |  |  | 3 | Not very confident |  |  |  |  |
|  |  |  | 4 | Not at all confident |  |  |  |  |
|  |  |  | 8 | Not Sure |  |  |  |  |
|  |  |  | 9 | Decline to answer |  |  |  |  |
| 113. | Do you perceive any barriers to mental health care access? | Mental Health | 1 | Yes | Quantitative | To Perceive | Health beliefs | Recipients |
|  |  |  | 2 | No |  |  |  |  |
|  |  |  | 0 | Don’t Know |  |  |  |  |
| 114. | What do you do when you have any of these conditions: [Probes: self-medication, health centre, pharmacy, traditional healer etc.]  o Fever  o Headache  o Cough/cold  o Diarrheal  o Wound or skin infection  o Severe illness  o A sick child | General Health Services / Primary Health Care | N/A | N / A | Qualitative | To Seek | Personal and social values | Recipients |
| 115. | What are healthcare options you seek whenever you are sick? | General Health Services / Primary Health Care | N/A | N / A | Qualitative | To Seek | Personal and social values | Recipients |
| 116. | Have you ever tried disease-screening tests, such as mammography or pap smear? | Maternal and Child Care | N/A | N / A | Qualitative | To Seek | Norms, | Recipients |
| 117. | Were there any other reasons that held you back from using maternal care services provided by healthcare professional (e.g. culture, religion, gender relationship in family)? | Maternal and Child Care | N/A | N / A | Qualitative | To Seek | Culture | Recipients |
| 118. | Does the support/lack of support from health personnel providing services have an influence on you in seeking HIV services? Please explain | Infectious Diseases / HIV/AIDS Health Services | N/A | N / A | Qualitative | To Seek | Personal and social values | Recipients |
| 119. | How would you describe your social relationship as a patient with doctors or nurses who serve you HIV services? | Infectious Diseases / HIV/AIDS Health Services | N/A | N / A | Qualitative | To Seek | Interpersonal Quality | Recipients |
| 120. | Does your social relationship with health professionals have an influence on you in seeking or accessing HIV /AIDS health services? Tell me more about it. | Infectious Diseases / HIV/AIDS Health Services | N/A | N / A | Qualitative | To Seek | Caregiver support | Recipients |
| 121. | Who makes decisions for you to visit the health-care service? | General Health Services / Primary Health Care | N/A | N / A | Qualitative | To Seek | Autonomy | Recipients |
| 122. | What can you tell about refugees who want to reach your care in the form of transport/language? | General Health Services / Primary Health Care | N/A | N/A | Qualitative | To Reach | Transport | Health Providers |
| 123. | How frequently do you use the health-care services? | General Health Services / Primary Health Care | N/A | N/A | Qualitative | To Reach |  | Recipients |
| 124. | Would you mind describing about your access to HIV/AIDS-related health services in your community? | Infectious Diseases / HIV/AIDS Health Services | N/A | N/A | Qualitative | To Reach | Accommodation | Recipients |
| 125. | How often do you access HIV/AIDS-related health services? Why? | Infectious Diseases / HIV/AIDS Health Services | N/A | Rarely/regularly/monthly | Qualitative | To Reach |  | Recipients |
| 126. | Is going to you nearest health facility convenient, or would you prefer something easier to reach? | General Health Services / Primary Health Care | N/A | Yes / No | Qualitative | To Reach | Geographic location | Recipients |
| 127. | What would you do if there is a particular medicine you are seeking but the facility/supplier doesn’t have it? | General Health Services / Primary Health Care | N/A | N / A | Qualitative | To Reach |  | Recipients |
| 128. | Do you think mothers in your community are able obtain the services they require without delays that put her life or health at risk? | General Health Services / Primary Health Care | N/A | N / A | Qualitative | To Reach |  | Recipients |
| 129. | Can you reach School Health Services professionals when you need them? | School Health Services | 1 | Never | Quantitative | To Reach |  | Health Providers |
|  |  |  | 2 | Sometimes |  |  |  |  |
|  |  |  | 3 | Often |  |  |  |  |
|  |  |  | 4 | Always |  |  |  |  |
|  |  |  | 5 | Not Applicable |  |  |  |  |
| 130. | Based on your experience, how easy would it be for you to get health care or advice from your clinic? | General Health Services / Primary Health Care | 1 | Not easy at all | Quantitative | To Reach | Social support | Recipients |
|  |  |  | Scale for 2, 3 and 4 not provided in the article. | |  |  |  |  |
|  |  |  | 5 | Very easy |  |  |  |  |
| 131. | How easy would it be for you to get medical advice from the clinic over the phone? | General Health Services / Primary Health Care | 1 | Not easy at all | Quantitative | To Reach | Social support | Recipients |
|  |  |  | Scale for 2, 3 and 4 not provided in the article. | |  |  |  |  |
|  |  |  | 5 | Very easy |  |  |  |  |
| 132. | What is your experience with having to pay for your health services? | General Health Services / Primary Health Care | N/A | N / A | Qualitative | To Pay |  | Recipients |
| 133. | What are the financial barriers/obstacles you face while utilizing the health-care services in your community? | General Health Services / Primary Health Care | N/A | N / A | Qualitative | To Pay |  | Recipients |
| 134. | Did you have to pay for maternal care services out-of-pocket? | Maternal and Child Care | N/A | Yes / No | Qualitative | To Pay | Health Insurance | Recipients |
| 135. | How much did you spend out-of-pocket in total (official + informal) for maternal care services during pregnancy, delivery and postnatal period? | Maternal and Child Care | N/A | N / A | Qualitative | To Pay | Income | Recipients |
| 136. | During your pregnancy and postnatal period (up to 42 days after birth) how many times did you NOT VISIT maternal care provider for any of the necessary services because you could not afford to pay either for the visit or for the transportation/travel? | Maternal and Child Care | N/A | N / A | Qualitative | To Pay | Income | Recipients |
| 137. | How much money do you spend on HIV/AIDS services and transport every time you access them? | Infectious Diseases / HIV/AIDS Health Services | N/A | N / A | Qualitative | To Pay | Income | Recipients |
| 138. | Did your household experienced a financial burden in order to pay for maternal care services? (e.g. had to TAKE or BORROW money from family, friends, bank, credit card) | Maternal and Child Care | N/A | N / A | Qualitative | To Pay | Social capital | Recipients |
| 139. | What sorts of resources do you spend on the costs of health services? | General Health Services / Primary Health Care | N/A | N / A | Qualitative | To Pay | Assets | Recipients |
| 140. | How do you cope with transport and health expenditures? | General Health Services / Primary Health Care | N/A | N / A | Qualitative | To Pay |  | Recipients |
| 141. | Do you have private health insurance to access healthcare services? | General Health Services / Primary Health Care | N/A | N / A | Qualitative | To Pay | Health Insurance | Recipients |
| 142. | Does physicians provide information about Zika risks/consequences to pregnancy during your pre-natal appointments? | Infectious Diseases / HIV/AIDS Health Services | N/A | Yes / No | Qualitative | To Engage | Information | Recipients |
| 143. | What is your experience with the patient– practitioner relationship? | General Health Services / Primary Health Care | N/A | N / A | Qualitative | To Engage | Empowerment | Recipients |
| 144. | During the past year, when you received care, has any (mental) health‐care professional you see for your condition discussed with you your main goals or priorities in caring for this condition? | Mental Health | 1 | Yes | Quantitative | To Engage | Empowerment | Recipients |
|  |  |  | 2 | No |  |  |  |  |
|  |  |  | 8 | Not Sure |  |  |  |  |
|  |  |  | 9 | Decline to answer |  |  |  |  |
| 145. | During the past year, when you received care, has any mental health‐care professional you see for your condition discussed with you your treatment options, including possible side effects? | Mental Health | 1 | Yes | Quantitative | To Engage | Empowerment | Recipients |
|  |  |  | 2 | No |  |  |  |  |
|  |  |  | 8 | Not Sure |  |  |  |  |
|  |  |  | 9 | Decline to answer |  |  |  |  |
| 146. | When you need care or treatment, how often does your regular GP or medical staff you see involve you as much as you want to be in decisions about your care and treatment? | General Health Services / Primary Health Care | 1 | Always | Quantitative | To Engage | Empowerment | Recipients |
|  |  |  | 2 | Often |  |  |  |  |
|  |  |  | 3 | Sometimes |  |  |  |  |
|  |  |  | 4 | Rarely or never |  |  |  |  |
|  |  |  | 5 | Not Applicable |  |  |  |  |
|  |  |  | 8 | Not Sure |  |  |  |  |
|  |  |  | 9 | Decline to answer |  |  |  |  |
| 147. | Did you feel like maternal care providers informed you sufficiently and tried to answer all your questions? | Maternal and Child Care |  | Yes | Quantitative | To Engage | Information | Recipients |
|  |  |  |  | No |  |  |  |  |
